# Deaths Involving Fentanyl, Fentanyl Analogs, and U-47700 — 10 States, July–December 2016

**DOI:** 10.15585/mmwr.mm6643e1

**Published:** 2017-11-03

**Authors:** Julie K. O’Donnell, John Halpin, Christine L. Mattson, Bruce A. Goldberger, R. Matthew Gladden

**Affiliations:** ^1^Division of Unintentional Injury Prevention, National Center for Injury Prevention and Control, CDC; ^2^Division of Forensic Medicine, Department of Pathology, Immunology and Laboratory Medicine, College of Medicine, University of Florida in Gainesville.

Preliminary estimates of U.S. drug overdose deaths exceeded 60,000 in 2016 and were partially driven by a fivefold increase in overdose deaths involving synthetic opioids (excluding methadone), from 3,105 in 2013 to approximately 20,000 in 2016 (*1*,*2*). Illicitly manufactured fentanyl, a synthetic opioid 50–100 times more potent than morphine, is primarily responsible for this rapid increase (*3*,*4*). In addition, fentanyl analogs such as acetylfentanyl, furanylfentanyl, and carfentanil are being detected increasingly in overdose deaths (*5*,*6*) and the illicit opioid drug supply (*7*). Carfentanil is estimated to be 10,000 times more potent than morphine (*8*). Estimates of the potency of acetylfentanyl and furanylfentanyl vary but suggest that they are less potent than fentanyl (*9*). Estimates of relative potency have some uncertainty because illicit fentanyl analog potency has not been evaluated in humans. This report describes opioid overdose deaths during July–December 2016 that tested positive for fentanyl, fentanyl analogs, or U-47700, an illicit synthetic opioid, in 10 states participating in CDC’s Enhanced State Opioid Overdose Surveillance (ESOOS) program.* Fentanyl analogs are similar in chemical structure to fentanyl but not routinely detected because specialized toxicology testing is required. Fentanyl was detected in at least half of opioid overdose deaths in seven of 10 states, and 57% of fentanyl-involved deaths also tested positive for other illicit drugs, such as heroin. Fentanyl analogs were present in >10% of opioid overdose deaths in four states, with carfentanil, furanylfentanyl, and acetylfentanyl identified most frequently. Expanded surveillance for opioid overdoses, including testing for fentanyl and fentanyl analogs, assists in tracking the rapidly changing illicit opioid market and informing innovative interventions designed to reduce opioid overdose deaths.

The 10 states^†^ reporting data abstracted information from preliminary death certificates and medical examiner/coroner reports on unintentional and undetermined opioid overdose deaths using standard definitions for variables. Data were entered into the State Unintentional Drug Overdose Reporting System (SUDORS), the component of ESOOS designed for tracking fatal opioid overdoses.^§^ For each death, available data on demographic characteristics, circumstances of the overdose collected from death scene investigations (e.g., evidence of illicit drug use), and results of forensic toxicology testing were entered into SUDORS. Opioid overdose deaths occurring during July–December 2016 with positive test results for fentanyl, fentanyl analogs, and U-47700 in 10 states are described, and key demographic and overdose circumstance factors are stratified by substance. Full toxicology findings of decedents were reviewed, including the presence of heroin, cocaine, and methamphetamine. Because heroin involvement in overdose deaths is difficult to distinguish from prescription morphine, deaths in which heroin was confirmed by toxicologic findings were combined with deaths in which heroin was suspected because morphine was detected and death scene evidence suggested heroin use.^¶^ The use of medical examiner/coroner reports, previously unavailable across states, provides unique insights into specific substances and circumstances associated with overdoses, which can inform interventions.

Fentanyl was detected in 56.3% of 5,152 opioid overdose deaths in the 10 states during July–December 2016 (Figure). Among these 2,903 fentanyl-positive deaths, fentanyl was determined to be a cause of death by the medical examiner or coroner in nearly all (97.1%) of the deaths. Northeastern states (Maine, Massachusetts, New Hampshire, and Rhode Island) and Missouri** reported the highest percentages of opioid overdose deaths involving fentanyl (approximately 60%–90%), followed by Midwestern and Southern states (Ohio, West Virginia, and Wisconsin), where approximately 30%–55% of decedents tested positive for fentanyl. New Mexico and Oklahoma reported the lowest percentage of fentanyl-involved deaths (approximately 15%–25%). In contrast, states detecting any fentanyl analogs in >10% of opioid overdose deaths were spread across the Northeast (Maine, 28.6%, New Hampshire, 12.2%), Midwest (Ohio, 26.0%), and South (West Virginia, 20.1%) (Figure) (Table 1).

**FIGURE:**
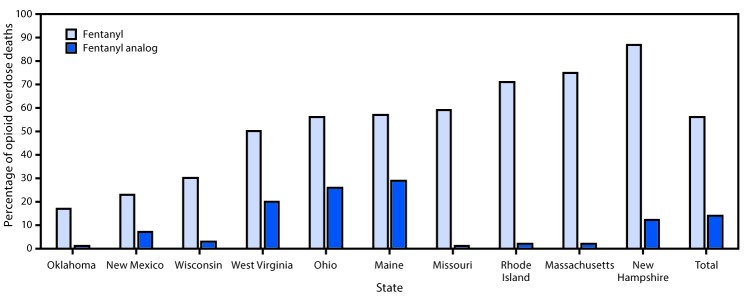
Percentage of opioid overdose deaths testing positive for fentanyl and fentanyl analogs, by state — 10 states, July–December 2016

**TABLE 1:** Number and percentage of opioid overdose decedents testing positive for fentanyl analogs and U-47700 — 10 states, July–December 2016

State	Total opioid overdose deaths	Any fentanyl analog present* No. (%)	Fentanyl analogs				U-47700 synthetic opioid No. (%)
			Carfentanil No. (%)	Furanylfentanyl No. (%)	Acetylfentanyl No. (%)	Other^†^ No. (%)	
**Total^§^**	**5,152**	**720 (14.0)**	**389 (7.6)**	**182 (3.5)**	**147 (2.9)**	**74 (1.4)**	**40 (0.8)**
Maine	**154**	**44 (28.6)**	0	25 (16.2)	17 (11.0)	5 (3.3)	—
Massachusetts	**1,071**	**17 (1.6)**	0	10 (0.9)	—^¶^	—	—
New Hampshire	**131**	**16 (12.2)**	0	—	13 (9.9)	0	—
New Mexico	**166**	**11 (6.6)**	0	—	7 (4.2)	0	—
Ohio	**2,043**	**531 (26.0)**	354 (17.3)	85 (4.2)	91 (4.5)	40 (2.0)	15 (0.7)
West Virginia	**393**	**79 (20.1)**	35 (8.9)	44 (11.2)	6 (1.5)	23 (5.9)	7 (1.8)
Wisconsin	**413**	**14 (3.4)**	0	6 (1.5)	5 (1.2)	—	5 (1.2)
Other three states**	**781**	**8 (1.0)**	0	—	—	—	—

* Individual fentanyl analog deaths might sum to a number greater than the number of deaths with any fentanyl analog present because more than one fentanyl analog could be present in an opioid overdose death.

^†^ Includes 3-methylfentanyl, acrylfentanyl, butyrylfentanyl, para-fluorofentanyl (or 4-fluorofentanyl), para-fluorobutyrylfentanyl (or 4-fluorobutyrylfentanyl), and para-fluoroisobutyrylfentanyl (or 4-fluoroisobutyrylfentanyl).

^§^ Data from 10 states included in the total numbers; individual states presented if five or more deaths tested positive for any fentanyl analog.

^¶^ Five or more deaths tested positive for acetylfentanyl in Massachusetts, but the number was suppressed to prevent calculation of number for other states, which was less than five.

** Missouri (22 counties), Oklahoma, and Rhode Island.

Fentanyl analogs were present in 720 (14.0%) opioid overdose deaths, with the most common being carfentanil (389 deaths, 7.6%), furanylfentanyl (182, 3.5%), and acetylfentanyl (147, 2.9%) (Table 1). Fentanyl analogs contributed to death in 535 of the 573 (93.4%) decedents. Cause of death was not available for fentanyl analogs in 147 deaths.^††^ Five or more deaths involving carfentanil occurred in two states (Ohio and West Virginia), furanylfentanyl in five states (Maine, Massachusetts, Ohio, West Virginia, and Wisconsin), and acetylfentanyl in seven states (Maine, Massachusetts, New Hampshire, New Mexico, Ohio, West Virginia, and Wisconsin). U-47700 was present in 0.8% of deaths and found in five or more deaths only in Ohio, West Virginia, and Wisconsin (Table 1). Demographic characteristics of decedents were similar among overdose deaths involving fentanyl analogs and fentanyl (Table 2). Most were male (71.7% fentanyl and 72.2% fentanyl analogs), non-Hispanic white (81.3% fentanyl and 83.6% fentanyl analogs), and aged 25–44 years (58.4% fentanyl and 60.0% fentanyl analogs) (Table 2).

**TABLE 2:** Demographic characteristics and overdose circumstance factors for decedents in opioid overdose deaths involving fentanyl, fentanyl analogs, and U-47700, by substance — 10 states, July–December 2016

Characteristic	Fentanyl (N = 2,903)	Any fentanyl analog* (N = 720)	Fentanyl analogs				U-47700 synthetic opioid (N = 40)
			Carfentanil (N = 389)	Furanylfentanyl (N = 182)	Acetylfentanyl (N = 147)	Other^†^(N = 74)	
	No. (%)	No. (%)	No. (%)	No. (%)	No. (%)	No. (%)	No. (%)
**Age group (yrs)** ^§^							
15–24	276 (9.5)	63 (8.8)	31 (8.0)	—^¶^	15 (10.2)	—	—
25–34	926 (31.9)	220 (30.6)	124 (31.9)	50 (27.5)	46 (31.3)	27 (36.5)	19 (47.5)
35–44	768 (26.5)	212 (29.4)	103 (26.5)	61 (33.5)	48 (32.7)	22 (29.7)	6 (15.0)
45–54	540 (18.6)	133 (18.5)	73 (18.8)	32 (17.6)	26 (17.7)	9 (12.2)	6 (15.0)
55–64	343 (11.8)	77 (10.7)	50 (12.9)	18 (9.9)	—	8 (10.8)	—
≥65	47 (1.6)	15 (2.1)	8 (2.1)	—	—	—	0
**Median age (IQR) in yrs**	37 (29–48)	38 (30–48)	39 (30–49)	38 (31–47)	36 (30–45)	36 (29–46)	32 (27–43)
**Sex**							
Male	2,080 (71.7)	520 (72.2)	276 (71.0)	134 (73.6)	111 (75.5)	49 (66.2)	32 (80.0)
Female	820 (28.2)	200 (27.8)	113 (29.0)	48 (26.4)	36 (24.5)	25 (33.8)	8 (20.0)
**Race and Hispanic origin**							
White, non-Hispanic	2,360 (81.3)	602 (83.6)	340 (87.4)	148 (81.3)	120 (81.6)	62 (83.8)	36 (90.0)
Black, non-Hispanic	274 (9.4)	75 (10.4)	42 (10.8)	17 (9.3)	9 (6.1)	9 (12.2)	—
Other, non-Hispanic	37 (1.3)	9 (1.3)	—	—	—	—	—
Hispanic	189 (6.5)	20 (2.8)	—	—	—	—	0
**Other fentanyl(s) present**							
Fentanyl or other fentanyl analog	n/a	330 (45.8)	120 (30.9)	93 (51.1)	143 (97.3)	46 (62.2)	24 (60.0)
Fentanyl	n/a	299 (41.5)	105 (27.0)	62 (34.1)	139 (94.6)	31 (41.9)	16 (40.0)
1 fentanyl analog present**	263 (9.1)	653 (90.7)	352 (90.5)	129 (70.9)	129 (87.8)	43 (58.1)	12 (30.0)
≥2 fentanyl analogs present	36 (1.2)	67 (9.3)	37 (9.5)	53 (29.1)	18 (12.2)	31 (41.9)	6 (15.0)
4-ANPP**^††^**	60 (2.1)	82 (11.4)	—	77 (42.3)	—	13 (17.6)	8 (20.0)
**Other illicit drugs present**							
Any illicit drugs	1,656 (57.0)	369 (51.3)	190 (48.8)	91 (50.0)	91 (61.9)	42 (56.8)	15 (37.5)
Suspected/Confirmed heroin^§§^	1,132 (39.0)	250 (34.7)	123 (31.6)	60 (33.0)	75 (51.0)	26 (35.1)	11 (27.5)
Cocaine	1,011 (34.8)	202 (28.1)	99 (25.4)	52 (28.6)	43 (29.3)	26 (35.1)	7 (17.5)
Methamphetamine	167 (5.8)	64 (8.9)	43 (11.1)	12 (6.6)	10 (6.8)	—	—
**Evidence of injection**	1,358 (46.8)	303 (42.1)	151 (38.8)	76 (41.8)	81 (55.1)	35 (47.3)	19 (47.5)
**No evidence of injection but evidence of other route** ^¶¶^	532 (18.3)	138 (19.2)	85 (21.9)	33 (18.1)	19 (12.9)	10 (13.5)	11 (27.5)
Evidence of snorting	279 (52.4)	95 (68.8)	57 (67.1)	21 (63.6)	15 (78.9)	9 (90.0)	8 (72.7)
Evidence of ingestion	203 (38.2)	41 (29.7)	27 (31.8)	8 (24.2)	7 (36.8)	—	—
Evidence of smoking	95 (17.9)	25 (18.1)	16 (18.8)	7 (21.2)	—	—	—
Evidence of transdermal	35 (6.6)	—	—	0	—	0	0
Evidence of sublingual	6 (1.1)	—	—	0	0	0	0
**No evidence of route**	1,013 (34.9)	279 (38.8)	153 (39.3)	73 (40.1)	47 (32.0)	29 (39.2)	10 (25.0)

**Abbreviation:** n/a = not applicable.

* Individual fentanyl analog deaths might sum to a number greater than the number of deaths with any fentanyl analog present because more than one fentanyl analog could be present in an opioid overdose death.

^†^ Includes 3-methylfentanyl, acrylfentanyl, butyrylfentanyl, para-fluorofentanyl (or 4-fluorofentanyl), para-fluorobutyrylfentanyl (or 4-fluorobutyrylfentanyl), and para-fluoroisobutyrylfentanyl (or 4-fluoroisobutyrylfentanyl).

^§^ Fewer than five persons aged ≤14 years died of an overdose that tested positive for a fentanyl analog.

^¶^ Data suppressed because fewer than five deaths, or suppressed to prohibit calculation of other suppressed cell.

** For fentanyl analogs, indicates no other analog present.

**^††^** Despropionylfentanyl is a fentanyl compound that can serve as a marker for illicitly manufactured fentanyl and fentanyl analogs because it is both a precursor and a metabolite of these illicit products (but not pharmaceutical fentanyl), while having low metabolic activity that does not contribute to overdose toxicity. Despropionylfentanyl is also known as 4-anilino-N-phenethylpiperidine, or 4-ANPP.

^§§^ Includes decedents testing positive for heroin metabolite 6-acetylmorphine, plus decedents testing positive for morphine where there was a history of heroin use, death scene evidence of illicit drug use, or evidence of injection, and no scene evidence of prescription drug use or other evidence of prescription morphine.

^¶¶^ Percentage of deaths with evidence of routes of administration other than injection calculated out of the number of deaths in this row.

Other illicit drugs co-occurred in 57.0% and 51.3% of deaths involving fentanyl and fentanyl analogs, respectively, with cocaine and confirmed or suspected heroin detected in a substantial percentage of deaths (Table 2). Nearly half (45.8%) of deaths involving fentanyl analogs tested positive for two or more analogs or fentanyl, or both. Specifically, 30.9%, 51.1%, and 97.3% of deaths involving carfentanil, furanylfentanyl, and acetylfentanyl, respectively, tested positive for fentanyl or additional fentanyl analogs. Forensic investigations found evidence of injection drug use in 46.8% and 42.1% of overdose deaths involving fentanyl and fentanyl analogs, respectively. Approximately one in five deaths involving fentanyl and fentanyl analogs had no evidence of injection drug use but did have evidence of other routes of administration. Among these deaths, snorting (52.4% fentanyl and 68.8% fentanyl analogs) and ingestion (38.2% fentanyl and 29.7% fentanyl analogs) were most common. Although rare, transdermal administration was found among deaths involving fentanyl (1.2%), likely indicating pharmaceutical fentanyl (Table 2). More than one third of deaths had no evidence of route of administration.

## Discussion

This analysis of opioid overdose deaths in 10 states participating in the ESOOS program found that illicitly manufactured fentanyl is a key factor driving opioid overdose deaths and that fentanyl analogs are increasingly contributing to a complex illicit opioid market with significant public health implications. Previous reports have indicated that use of illicitly manufactured fentanyl mixed with heroin, with and without users’ knowledge, is driving many fentanyl overdoses, particularly east of the Mississippi River (*3*,*4*). Consistent with these findings, at least half of opioid overdose deaths in six of the seven participating states east of the Mississippi tested positive for fentanyl. Over half the overdose deaths involving fentanyl and fentanyl analogs tested positive for confirmed or suspected heroin (the most commonly detected illicit substance), cocaine, or methamphetamine. This supports findings from other reports indicating that fentanyl and fentanyl analogs are commonly used with or mixed with heroin or cocaine (*3*,*4*). Nearly half of overdose deaths involving fentanyl and fentanyl analogs, however, did not test positive for other illicit opioids, suggesting that fentanyl and fentanyl analogs might be emerging as unique illicit products.

Fentanyl and fentanyl analogs are highly potent and fast-acting synthetic compounds that can trigger rapid progression to loss of consciousness and death and thus might require immediate treatment and high doses of naloxone (*5*). Because of the potency of fentanyl and fentanyl analogs and the rapid onset of action, these drugs were determined by medical examiners and coroners to play a causal role in almost all fatal opioid overdoses in which they were detected. Injection, the most commonly reported route of administration in fatal overdoses, exacerbates these risks because of rapid absorption and high bioavailability. The high potency of fentanyl and fentanyl analogs, however, can result in overdose even when administered via other routes. Nearly one in five deaths involving fentanyl and fentanyl analogs had evidence of snorting, ingestion, or smoking, with no evidence of injection. Multiple overdose outbreaks and law enforcement drug product submissions across the country have reported counterfeit prescription pills laced with fentanyl and fentanyl analogs (*10*).

With few exceptions, fentanyl analogs are illicitly manufactured, because they do not have a legitimate medical use in humans.^§§^ The detection of fentanyl analogs in >10% of opioid overdoses in four states raises the concern that fentanyl analogs have become a part of illicit opioid markets in multiple states. The fentanyl analogs most commonly detected were carfentanil, furanylfentanyl, and acetylfentanyl. Carfentanil, which is intended for sedation of large animals, is much more potent than fentanyl, whereas furanylfentanyl and acetylfentanyl are less potent (*9*). Carfentanil contributed to approximately 350 overdose deaths in Ohio, but was detected in only one other state (West Virginia). Because of its extreme potency, even limited circulation of carfentanil could markedly increase the number of fatal overdoses. Recent data suggest that carfentanil deaths are occurring in multiple other states, including Kentucky, which reported 10 overdose deaths involving carfentanil in the second half of 2016 (Kentucky Department of Public Health, unpublished data, 2017) and New Hampshire, which reported 10 deaths in 2017.^¶¶^ Forty-six percent of SUDORS opioid overdose deaths involving fentanyl analogs tested positive for fentanyl or an additional fentanyl analog, ranging from 31% for carfentanil to 97% for acetylfentanyl. The increased mixing or co-use of fentanyl, heroin, cocaine, and varying fentanyl analogs might contribute to increased risk for overdose because persons misusing opioids and other drugs are exposed to drug products with substantially varied potency.

The findings in this report are subject to at least five limitations. First, results are limited to 10 states and therefore might not be generalizable. Second, the presence of fentanyl analogs is underestimated because commonly used toxicologic testing does not include fentanyl analogs, some fentanyl analogs are difficult to detect (*9*), and specialized testing for fentanyl analogs varied across states and over time. Third, the route of fentanyl and fentanyl analog administration must be interpreted cautiously because the data do not link specific drugs to routes of administration and thus the precise route of administration of fentanyl or fentanyl analogs cannot be determined in overdose deaths involving multiple substances (e.g., heroin and cocaine) and routes (e.g., injection and snorting). Fourth, the combination of deaths with toxicologic confirmation of heroin with those with detection of morphine and death scene evidence suggesting heroin use might have resulted in misclassification of some deaths. Finally, fentanyl source could not be definitively determined; however, only a small percentage of fentanyl deaths had evidence consistent with prescription fentanyl (e.g., transdermal use versus injection).

Illicitly manufactured fentanyl is now a major driver of opioid overdose deaths in multiple states, with a variety of fentanyl analogs increasingly involved, if not solely implicated, in these deaths. This finding raises concern that in the near future, fentanyl analog overdose deaths might mirror the rapidly rising trajectory of fentanyl overdose deaths that began in 2013 and become a major factor in opioid overdose deaths. In response to this concern, CDC expanded ESOOS to 32 states and the District of Columbia in 2017 and added funding for all 33 recipients to improve forensic toxicologic testing of opioid overdose deaths to include capacity to test for a wider range of fentanyl analogs.*** Increased implementation of evidence-based efforts targeting persons at high risk for illicit opioid use, including increased access to medication-assisted treatment, increased availability of naloxone in sufficient doses, and other innovative intervention programs targeting this group, is needed to address a large and growing percentage of opioid overdose deaths involving fentanyl and fentanyl analogs.

**Summary**

**What is already known about this topic?**

Sharp increases in opioid overdose deaths since 2013 are partly explained by the introduction of illicitly manufactured fentanyl into the heroin market. Outbreaks related to fentanyl analogs also have occurred. One fentanyl analog, carfentanil, is estimated to be 10,000 times more potent than morphine. Fentanyl analogs are not routinely detected because specialized toxicology testing is required.

**What is added by this report?**

This is the first report using toxicologic and death scene evidence across multiple states to characterize opioid overdose deaths. Fentanyl was involved in >50% of opioid overdose deaths, and >50% of deaths testing positive for fentanyl and fentanyl analogs also tested positive for other illicit drugs. Approximately 700 deaths tested positive for fentanyl analogs, with the most common being carfentanil, furanylfentanyl, and acetylfentanyl.

**What are the implications for public health practice?**

Increasing mixing or co-use of fentanyl, heroin, cocaine, and fentanyl analogs might contribute to increased overdose risk, because users are exposed to drug products that vary substantially in potency and that include some extremely potent products. Surveillance for opioid overdoses needs to expand to track the rapidly changing illicit opioid market. In fall 2017, CDC funded 33 jurisdictions to expand forensic toxicology testing. Increased implementation of evidence-based efforts targeting persons at high risk for using illicit opioids, including increased access to medication-assisted treatment and increased availability of naloxone, and innovative interventions are needed.

## Footnotes

* CDC’s Enhanced State Opioid Overdose Surveillance program funded 12 states through a competitive application process in fiscal year 2016. Data were available for this report for 10 of the 12 states. https://www.cdc.gov/drugoverdose/foa/state-opioid-mm.html.

† Maine, Massachusetts, Missouri (data available for 22 counties), New Hampshire, New Mexico, Ohio, Oklahoma, Rhode Island, West Virginia, and Wisconsin

§ State Unintentional Drug Overdose Reporting System (SUDORS) estimates of opioid-involved overdose deaths might differ from those of the National Vital Statistics System because SUDORS uses preliminary death certificate data and collects additional information from medical examiner and coroner reports, which are abstracted within 8 months of death. In SUDORS, an opioid-involved overdose death either was identified through review of the medical examiner/coroner report or had *International Classification of Disease, Tenth Revision* (ICD-10) underlying cause-of-death codes X40–44 (unintentional) or Y10–Y14 (undetermined) and multiple cause-of-death codes T40.0, T40.1, T40.2, T40.3, T40.4, or T40.6 on the death certificate. Data for this report were downloaded on September 5, 2017.

¶ A confirmed heroin death is defined as a death that tested positive for the heroin metabolite 6-acetylmorphine. The heroin metabolite 6-acetylmorphine, however, rapidly metabolizes to morphine, and thus a death involving heroin might only test positive for morphine, which is also present in deaths involving prescription morphine. A suspected heroin death is one in which testing for morphine is positive and the decedent also has a history of heroin use or death scene evidence indicating illicit drug use or injection in the absence of any evidence of prescription drug use or use of prescription morphine. https://www.ncbi.nlm.nih.gov/pubmed/25041514.

** Illicitly manufactured fentanyl is more easily mixed with white powder heroin, which is primarily sold east of the Mississippi River, than with black tar heroin. Although white powder heroin dominates the heroin market in the Northeast, the heroin market in Missouri includes both white powder heroin and black tar heroin. This might, in part, explain the high percentage of fentanyl overdoses documented in the state. Additional information available at https://www.justice.gov/archive/ndic/dmas/Midwest_DMA-2011(U).pdf.

†† Data on whether fentanyl analogs contributed to the death in which they were detected was not available for 20.4% of deaths with fentanyl analogs. As new fentanyl analogs emerged, they were captured as free text (without the option to indicate whether they contributed to the death) until being added to the menu of substances in the toxicology portion of SUDORS.

§§ The three fentanyl analogs with legitimate human medical use are remifentanil, alfentanil, and sufentanil; none of the SUDORS deaths was positive for these substances. Carfentanil is used exclusively in large animal veterinary medicine.

¶¶ Additional information is available at https://www.dhhs.nh.gov/dcbcs/bdas/documents/dmi-august-2017.pdf.

*** https://www.cdc.gov/drugoverdose/foa/state-opioid-mm.html.
